# Children's own perspectives demonstrate the need to improve paediatric perioperative care

**DOI:** 10.1002/nop2.332

**Published:** 2019-07-18

**Authors:** Gunilla Lööf, Nina Andersson‐Papadogiannakis, Charlotte Silén

**Affiliations:** ^1^ Department of Learning, Informatics, Management and Ethics Karolinska Institutet Stockholm Sweden; ^2^ Department of Paediatric Anaesthesia and Intensive Care Karolinska University Hospital Stockholm Sweden; ^3^ Department of Women's and Children's Health Karolinska Institutet Stockholm Sweden

**Keywords:** anaesthesia, children, communication, education and practice development, information needs

## Abstract

**Aim:**

To explore children's perspectives when facing anaesthesia and surgery.

**Design:**

Interpretative qualitative design.

**Methods:**

Children undergoing outpatient surgery were interviewed in three different phases, before and after anaesthesia and surgery (phase 1 and 2) and 1 month after the hospitalization (phase 3). Twenty‐two children (4–15 years) were interviewed in phase 1 and 2 and six children (5–13 years) in phase 3. Data were analysed using manifest and latent qualitative content analysis.

**Results:**

Two contrasting themes were identified: Fearful in association with anaesthesia and surgery and Confident in association with anaesthesia and surgery. Comprehension of the perioperative procedures, continuous information and interaction with the healthcare providers were decisive factors for children's expressions of confidence or fearfulness. Events considered as major from a healthcare perspective may be of no importance to the child, while events considered as less important by healthcare providers may be significant to the child. Understanding children's perspectives and awareness of their need to process the information provided are significant factors for establishment of trust and confidence in a highly technological perioperative environment.

## 1 INTRODUCTION

Anaesthesia and surgery are significant and stressful experiences for hospitalized children (Coyne, 2006; Pelander & Leino‐Kilpi, 2010) which incorrectly handled may affect treatment, recovery and future dealings with medical services (Kain, Mayes, Caldwell‐Andrews, Karas, & McClain, 2006; Kain, Mayes, O'Connor, & Cicchetti, 1996; Karling, 2006). Uncertainty of procedures, fear for pain and separation from parents are all sources of preoperative anxiety affecting a significant number of children undergoing anaesthesia and surgery (Coyne, 2006; Kain et al., 1996; Wilson, Megel, Enenbach, & Carlson, 2010). Variables such as the child's age and cognitive ability, temperament, previous medical experiences, attitudes of the healthcare providers and anxiety of the parent have been identified as predictors of preoperative anxiety resulting in increased manifestations of general anxiety, agitation, increased pain and analgesic consumption during the early postoperative period (Kain et al., 2006, 1996). Many children also show late reactions such as apathy, temper tantrums, eating disorders, fear of death, separation anxiety and sleeping problems during the weeks following anaesthesia and surgery (Kain et al., 2006; Karling, 2006). A growing body of evidence highlights the importance of paying attention to children's experiences of a hospitalization (Carney et al., 2003; Coyne & Kirwan, 2012) but few studies are related to children's view of anaesthesia and surgery (Fortier et al., 2009; Smith & Callery, 2005).

### 1.1 Background

Children are competent to self‐report their feelings, thoughts and opinions (Carney et al., 2003; Coyne, 2006, 2008) and can participate in research processes as long as the researcher recognizes the way they communicate and facilitates their participation (Kirk, 2007). Current research regarding children's understanding of anaesthesia and surgery mostly reports beliefs of healthcare professionals and parents without addressing children's own experiences. There is an increasing awareness that children's perceptions and experiences differ from adults, thus adults acting as proxies for children is not sufficient (Nilsson et al., 2015; Söderbäck, Coyne, & Harder, 2011). A child perspective is based on an external view of children's conditions, experiences, feelings and actions, usually with the child's best interest in mind. A child's perspective is characterized by what the child finds important. The adult's proxy perspective means showing an interest in the child's world and what is best for them whereas to understand the child's perspective means listening to and considering children's voices (Sommer, Samuelsson, & Hundeide, 2009). There is a growing international recognition that children have the right to participate in matters affecting their lives (Coyne, 2008). Children's right to express themselves and receiving information is important for their participation (Coyne, 2008; Sjöberg, Amhliden, Nygren, Arvidsson, & Svedberg, 2015) reduction of stress and for enhancing understanding and adaption (Coyne & Kirwan, 2012).

Despite a wide base of evidence proclaiming the role of preparation of children for anaesthesia and surgery (Fortier et al., 2009; Jaaniste, Hayes, & Baeyer, 2007; Kain & Caldwell‐Andrews, 2005), many continue to report lack of information and preparation (Coyne & Kirwan, 2012; Smith & Callery, 2005). Studies show that children want to get information to understand their illness and be involved, consulted and heard in relation to their informational needs (Coyne, 2008; Coyne & Kirwan, 2012). However, studies of children's perspectives of the perioperative processes are still relatively uncommon (Jaaniste et al., 2007; Kassa, Engvall, & Engstrand Lilja, 2017; Sjöberg et al., 2015). Identification of what information is desired by children is vital for the design of preoperative preparation programmes and for care of the child during the perioperative processes. Even though it has been shown that children can identify their needs for preparation and contribute to the development of preoperative preparation programmes, their involvement in the developmental processes is rare (Fortier et al., 2009; Smith & Callery, 2005). We argue that children's voices are needed to optimize understanding and cooperation as well as to reduce preoperative anxiety and postoperative trauma. Based on the research question on how children express their experiences in relation to different stages of the perioperative period, the aim of this study was to explore children's perspectives when facing anaesthesia and surgery.

### 1.2 Design

This study adopted a qualitative approach to obtain understanding of children's perspectives when facing perioperative care (Krippendorff, 2012; Patton, 2015). Qualitative methods can be used in research with children at various developmental stages to provide an insight into their experiences, understanding and perceptions by collection of self‐expressed views (Kortesluoma, Hentinen, & Nikkonen, 2003).

## 2 METHODS

### 2.1 Data collection

**Figure 1:**
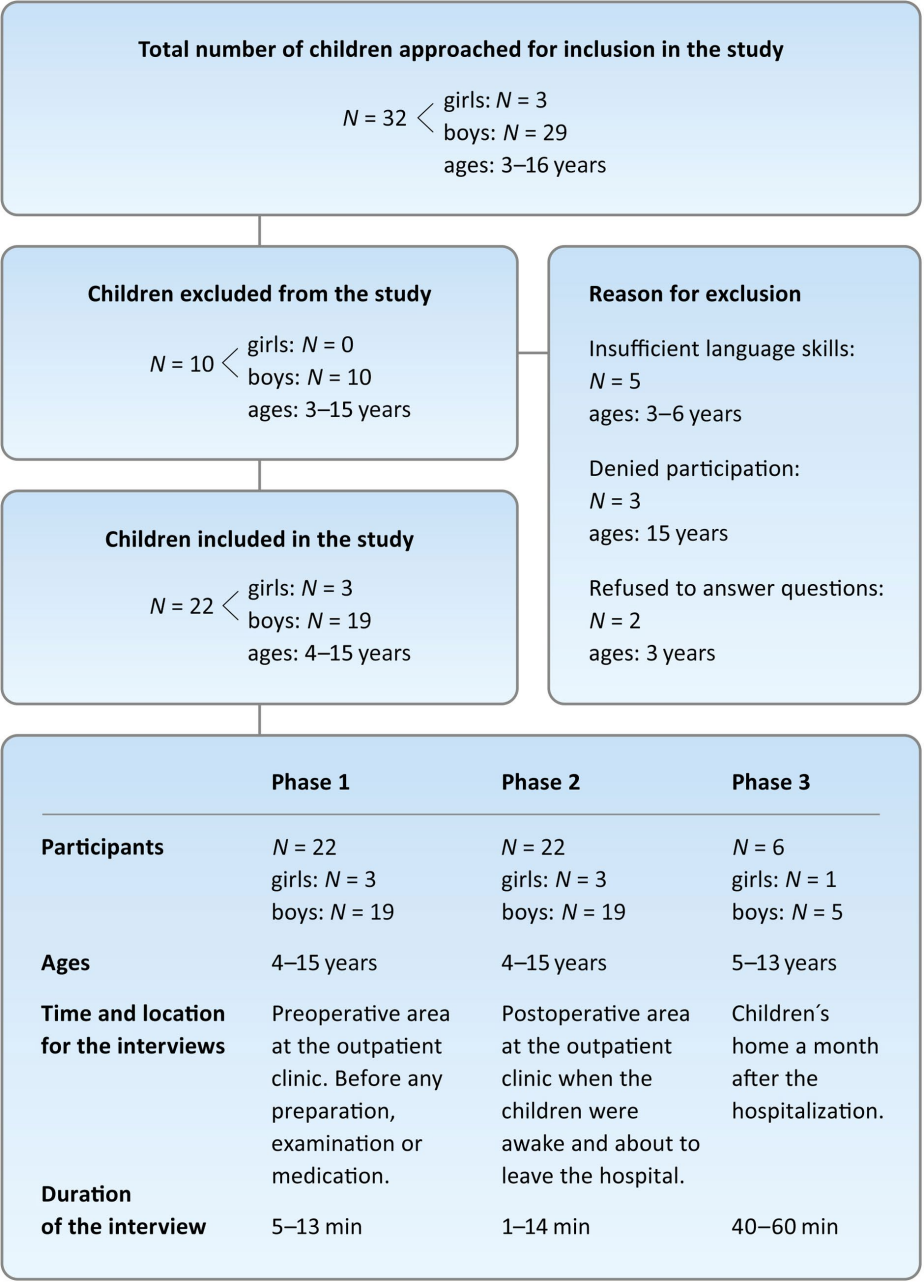
Overview of the participants and interviews conducted

Interviews were performed in three different phases. All families with children 3–16 years of age (*N* = 32) admitted for outpatient surgery were asked to participate. Children were interviewed before (phase one) and after (phase two) anaesthesia and surgery and at home 1 month after the hospitalization (phase three; Figure 1). The families visited the preoperative assessment clinic a month before admittance, received information from an anaesthesiologist and were advised to take part of written information and visit the Anesthesia Web (www.anaesthesiaweb.org). The families were asked to participate on arrival at the hospital. The request was followed by verbal and age differentiated written information piloted with children of different ages.

Twenty‐two children (4–15 years) were interviewed before and after anaesthesia and surgery. All were asked to participate in follow‐up interviews a month after the hospitalization. Sixteen accepted and six children (5–13 years) representing a range of ages, experiences and personalities were chosen to participate in phase 3 (Figure 1). The parent was present at the interviews at the hospital but not during the interviews in the home.

The semi‐structured interviews followed an interview guide based on areas about children's experiences of hospitalization and need for preparation (Appendix S1). A nurse specializing in anaesthesia and paediatrics (GL), with extensive experience of preparing families for medical procedures, performed all interviews. The interviewer had no previous contact with the families and was not informed about the child's medical condition.

### 2.2 Data analysis

The interviews were transcribed verbatim indicating non‐verbal communication, such as silences, looks, laughter and gestures (Irwin & Johnson, 2005). A qualitative content analysis primarily guided by Krippendorff (2012) and Graneheim and Lundman (2004) was applied. The *manifest content* refers to the visible and obvious components answering the question ‘What?’, thought of as ‘what the child says.’ The *latent content* answers the question ‘Why?’, notes the underlying meaning of children's expressions and ‘what the text is talking about'. The analysis of the text was performed in several steps by a research team representing extensive knowledge within the areas of paediatrics, anaesthesia, medical education, preoperative and web‐based information. The transcripts were read several times to ensure familiarity with the data and to get a sense of the whole. Meaning units were identified, condensed and labelled with a code. Codes that deviated from the aim were excluded, and the remaining codes were sorted in to twelve categories at a manifest level. Finally, the underlying meaning of the categories was interpreted; two themes and four sub‐themes were identified based on consensus in the research group. All themes were supported by data from children of different ages and with different experiences. Although some children tended to respond just with one or two short sentences, these provided a rich insight into their own thoughts and experiences. The research team continuously returned to the transcribed text to ensure that the analysis and the coding accurately reflected what the children stated and to confirm the consistency of the themes.

### 2.3 Ethics

The study was approved by the Regional Ethical Review Board in Stockholm. Informed and written consent were obtained from both parents and children. Developmental limitations, the imbalance of power between children, parents and healthcare providers were taken into consideration throughout the research process. Both children and parents were informed that consent was negotiable and possible to withdraw at any point.

## 3 RESULTS

**Figure 2:**
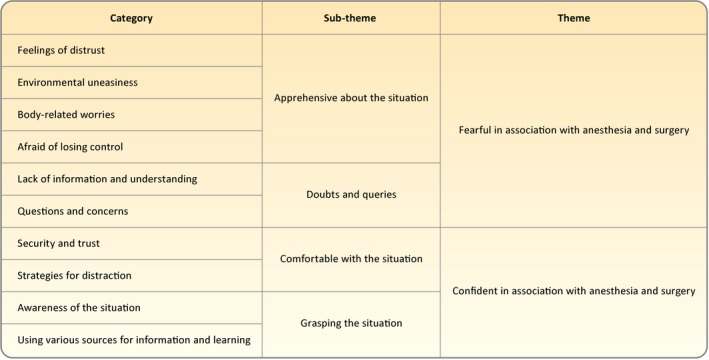
Overview of the themes, sub‐themes and categories describing children's experiences when facing anaesthesia and surgery

Interpretation of children's perspectives when facing anaesthesia and surgery resulted in two contrasting themes: *Fearful* in association with anaesthesia and surgery and *Confident* in association with anaesthesia and surgery. Children's experiences of fearfulness rest on two sub‐themes: *Apprehensive about the situation* and *Doubts and queries* whereas children's experiences of confidence rest on the sub‐themes *Comfortable with the situation* and *Grasping the situation*. The sub‐themes within each of the themes are connected. *Doubts and queries* relates to experiences of being *Apprehensive about the situation*, while *Grasping the situation* relates to experiences of being *Comfortable with the situation* (Figure 2).

### 3.1 Fearful in association with anaesthesia and surgery

#### 3.1.1 Apprehensive about the situation

Children's expressions of being apprehensive about the situation were multifaceted and characterized by *Feelings of distrust, Environmental uneasiness, Body‐related worries and Afraid of losing control*. Previous negative experiences of anaesthesia and surgery increased children's fears.

*Feelings of distrust* were related to interactions with the medical providers and to the information received. Increased distrust were voiced when children's experiences not were matching received information and expectations: ‘I don't trust people in the hospital, they are lying (Child 9, Boy 5y). The doctor said it would go quick and won't hurt since my skin was sleeping, but it was so painful and they continued forever’ (Child 14, Boy 11y). The communication with the medical providers was pointed out as another source of distrust: ‘I didn't understand what they said. They held me hard and continued with the painful procedures without listening even though I cried out loudly’ (Child 9, Boy 5y).

*Environmental uneasiness* was mostly related to the operating room: ‘I got really anxious when I entered the operating room and saw all the scary things’ (Child 22, Girl 11y). The operating room was experienced as cold, strange smelling and filled with frightening, technical equipment. Children described the hard and narrow operating table and the operating lamp shining right in their eyes. They also referred to the odour from the anaesthesia mask and the sound of the anaesthesia suction. Teenagers mentioned the amount of people in the operating room. They questioned what everyone was doing and experienced a crowded room as stressful.

In terms of *body‐related worries*, the needle‐related procedures were most central. Children recalled previous negative experiences and were afraid to experience the same. The removal of the patch with the local anaesthetic cream was experienced as almost as bad as having the intravenous access placed. When children pictured treatments and investigations, they expressed worries and concerns for mistakes being made. ‘Someone will cut in me. I really hope they can handle sharp knives and will do everything right’ (Child 7, Boy 12y). Worries for an altered body image and the risk for visible scars or lifelong punishment were also highlighted. One child expressed how the surgeon had done the preoperative marking on his shoulder when the surgery was planned for removal of some small pins at his wrist. ‘I was scared to death and thought they were going to operate at the wrong place or amputate my arm. I cried and was almost vomiting but was too scared to explain for anyone why’ (Child 11, Boy 9y).

A central source for worry by teenagers was the discomfort of having a stranger affect their physical integrity: ‘Even though the health care providers say they don't care they have to understand it is a strange feeling for me to expose my body for strangers’ (Child 5, Girl 13y). ‘Strangers will handle my willie when I am sleeping, I am wondering what they will be thinking’. (Child 7, Boy 12y). Children's body‐related experiences were further related to being hungry due to the preoperative eating restrictions and the unpleasant, overwhelming and frightening experience of waking up with pain, nausea, headache and dizziness. Another prominent source for worry was the unexpected hoarseness and longs‐lasting feeling of having a sore throat.

Being *afraid of losing control* was described in regard to the physical and psychological mechanisms and to the loss of control of daily structures and context. Fear of losing control were described in relation unconsciousness during anaesthesia: ‘It was such a scary feeling to lose control whey I received the sleeping medicine. Everything happened so extremely fast and I wasn´t prepared for it’ (Child 22, Girl 11y); *for the risk of waking up during the surgery* ‘I was not really so afraid to wake up during the surgery since I know the security is so high, but anyway, I was thinking of the risk of it all the time since everything that happened was out of my control’ (Child 3, Boy 14y) and for not waking up after the procedure ‘I was so scared I would be asleep forever and never wake up again’ (Child 5, Girl 13y). Children were afraid of the risk that the medication given to start off the anaesthesia would have a reduced effect if the surgery was prolonged and the risk for that the oxygen might run out. Children's medical references were often associated with impressions from media: ‘I saw a television (TV) documentary where they operated on a lion who woke up before the surgery was done’ (Child 20, Boy, 11y) and ‘I have seen how the monitors can induce an electric chock’ (Child 5, Girl 13y). Losing control of daily structures and context was also described: ‘Time will go so fast and disappear. I will miss a lot of things going on with my friends’ (Child 21, Boy 15y).

#### 3.1.2 Doubts and queries

Children's voices about reasons for fear were illuminated by their *Lack of information and understanding* and unanswered or disclosed *Questions and concerns.*

A general *lack of information and understanding* about the cause for hospitalization and procedures were mentioned as the main reasons for being apprehensive. Children were lacking information and expressed questions about procedures and their possible outcomes, the hospital environment, timing of different perioperative phases, discharge procedures and postoperative medical conditions. Throughout the interviews, children lacked the ability to put individual events in a broader perspective and understand their significance in the context. They could explain what should be done, but not the reasons for why: ‘If I eat before the surgery the surgeon will have problems while operating in my body’ (Child 16, Boy 10y), ‘The anesthesia won't have full effect if I eat since all systems in my body will be focused on the food digestion’ (Child 5, Girl 13y).

The needle‐related procedures were associated with most *questions and concerns* in all interviews. Children expressed concerns for the needle from the intravenous access to remain in the blood vessel: ‘I thought the needle would injure other parts of my hand if I was moving. I became so relieved when a nurse explained there was only a small plastic tube remaining’ (Child 14, Boy 11y). The use of medical terminology, fictive and technological words were another source for questions and concerns. It was obvious that common used terms were misunderstood and had different meaning for children and healthcare providers.

### 3.2 Confidence in association with anaesthesia and surgery

#### 3.2.1 Comfortable with the situation

Children's expressions about being comfortable were characterized by: *Feelings of security and trust* and *Strategies for distraction*. Their expressions were mostly related to positive experiences despite the fact that facing anaesthesia and surgery was described as challenging.

*Security and trust* were related to the positive interactions with the healthcare providers and understanding of the information received. Children expressed how friendly, reassuring descriptions, openness for questions and understandable explanations were reducing their feelings of distress. They highlighted the importance of receiving information in good time as well as a continuous information during all perioperative phases: ‘When you are nervous it’s difficult to remember, you have to hear it many times to catch it’ (Child 7, Boy 12y).

The anaesthesia providers description of the monitoring and conviction of their constant presence during the surgery contributed to feelings of security and trust: ‘The monitors measured my heart rate and breathing. When my heart rate went up they knew I had pain and was given me more medicine’ (Child 7, Boy 12y). ‘They had total control, someone, who was not even allowed go to the toilet, was sitting by my side checking me and the monitors’ (Child 1, Boy 8y). Expressed as important was the opportunity to control small matters, such as where to insert the intravenous access, on what finger to set the oxygen probe, holding the anaesthesia mask and being a part of the decision when to start the anaesthesia. The assurance of the presence of parents during all perioperative phases was of main importance for children's feeling secure and trust: ‘I felt calm and safe as my mother was present all the time. She was with me holding my hand both when I was putting asleep and was wakening up. She also explained things I didn´t understood’ (Child 20, Boy 11y).

Children portrayed various strategies for distraction to cope with the stressful situation. Distraction was searched by asking for information, support and security from adults. Creating positive images, thinking about fun, focusing on learning and telling themselves to accept the situation were also described as deliberate strategies. The allowance of toys and cell phones was emphasized as important for distraction. By playing with their toys, watching a film and keeping busy in chatting with friends on social media they were able to distance themselves from what they were facing. Children appreciated the anaesthesia provider's strategies for distraction when talking about daily activities, telling a story and having humour. They also valued the hospital clowns and the rewards received after surgery.

#### 3.2.2 Grasping the situation

Children's experience of confidence was related to their feelings of grasping the situation. This sub‐theme is characterized by children expressing *Awareness of the situation* and *Using various sources for information and learning.* The confident children displayed an *awareness of the situation*, understanding about preparations and procedures and were able to situate them in the context of the perioperative processes.

Children's awareness were exemplified by understanding of the use of the local anaesthetic cream for reducing pain and removal of the needle from the intravenous access: ‘Even though they call it for a needle I have learned that there is only a small plastic tube remaining in the blood vessel’ (Child 4, Boy 8y). The intravenous access was explained as a connection to the blood vessels helping the heart to distribute medications and fluids in the body: ‘The sleeping medication I received was spread via the blood vessels up in my head affecting areas for sleep and alertness’ (Child 7, Boy 12y); ‘I continued getting sleep and pain medication throughout the surgery’ (Child 20, Boy 11y). Children's understanding of the fasting routines was described as ‘Since the throat is totally relaxed during anesthesia there is a risk for vomiting and aspiration of food to the lungs. This is something you can die from’ (Child 18, Boy 13y).

Children described how they were *using various sources for information and learning* to get information about anaesthesia and surgery. Parents, specifically their mothers, were the ones who had mainly provided them with information. They also referred to doctors and nurses as having a central role in providing them with information. Nevertheless, they did not feel comfortable asking the healthcare providers questions, and they preferred asking their parents. Other sources to get information associated with the preoperative visits were described as reading complementary printed information material and visiting a web‐based preparation programme.

## 4 DISCUSSION

In line with earlier studies (Kassa et al., 2017; Sjöberg et al., 2015), children voiced anaesthesia and surgery as significant events. Their messages need to be taken seriously since understanding of their perspectives is crucial for healthcare providers to support and assure trust and safety during the perioperative processes.

As can be expected, the specificity and diversity of responses increased with age. Young children's responses were short, simple and concrete in the moment reflecting their limited expressive and cognitive abilities. School‐aged children were able to verbalize their experiences and made suggestions of improvement and teenagers demonstrated abstract thinking. However, despite an individual variation and age‐different ways of conveying their experiences, it was obvious that children's experiences to a high extent were similar regardless of age, gender, diagnoses and previous medical experiences.

Children's voices unfolded two contrasting themes: *Fearful* in association with anaesthesia and surgery and *Confident* in association with anaesthesia and surgery. Although the themes to a great extent reflected children's opposite experiences similarities were found, common features were related to children's perspectives compared with the expectations of adult's and healthcare providers. Children noticed and perceived far more and other things during the perioperative process than might be expected by the healthcare providers. The hospitalization was not perceived from a traditional perioperative perspective including a pre‐, per‐ and postoperative phase. Instead, children encountered one big significant event including a variety of smaller and bigger happenings. Events considered as major from a healthcare perspective may be of no importance to the child, while events considered as less important may be most significant to the child. The perioperative environment caught attention both in regard to the visible, audible and smelling surroundings as well as to actions and attitudes.

Considering the differences exposed in the two contrasting themes, important messages were displayed. Overall, the confident children described an awareness of the situation, preparations and procedures in the context of illness and need for anaesthesia and surgery. The opposite was the case for children displaying fearfulness. Their experiences were related to not being prepared for the expected procedures, and they were lacking understanding of context for procedures and the terminology used. Children's messages about the importance of comprehension about the situation and the interrelatedness to communication and trust in healthcare providers are confirmed by previous studies. Moore and Kirk (2010) described barriers for children's participation in communication with healthcare professionals, including language difficulties, lack of necessary medical skills and thereby an inability to ask appropriate questions. Deficiency of information causing increased concern and lack of understanding when children encounter unexpected experiences has also been reported (Bray, Callery, & Kirk, 2012; Sjöberg et al., 2015).

The needle‐related procedures came to the fore as central for all children and this is consistent with previous studies about children's fears in hospital (Fortier, Rosario, Martin, & Kain, 2010; Pelander & Leino‐Kilpi, 2010). Unexpected was that the removal of the tape covering the anaesthetic cream was described as worse than getting the intravenous access. The fearful children expressed apprehension about pain and potential risks for mistakes in relation to surgery. On the contrary, the confident children were capable to relate symptoms and experiences to anatomical and physiological knowledge and the performed preparations and procedures.

As previously described (Kain, Wang, Mayes, Caramico, & Hofstadter, 1999), the entrance to the operating room was a stressful part of the perioperative processes. Apprehensive children were frightened by the aseptic smell, sounds from monitors and too many loud talking people repeatedly asking the same questions. Instead, the confident children conveyed a curiosity and an opportunity to learn new things and conveyed trust and confidence to receive help whenever needed. Experiences were also affected by how they perceived their interaction with the healthcare providers. While the confident children expressed well function interaction and trust, the fearful children voiced distrust and lack of communication.

Healthcare providers behaviour and attitudes have been described as influencing children's experiences of stress (Chorney, Tan, & Kain, 2013). Allowing children to voice their opinions and ask questions can make a significant difference in their wellbeing (Coyne & Kirwan, 2012) as well for the effectiveness and outcome of the perioperative procedures (Fortier et al., 2009; Sjöberg et al., 2015). Previous experiences of anaesthesia and surgery emerged as a poor protection for preoperative anxiety. Instead, these children showed an extended need of preparation. In contrast to the often‐occurring attitude of adults to protect children, by limiting the information provided, children in line with previous investigations (Buckley & Savage, 2010; Fortier et al., 2009; Jaaniste et al., 2007) asked for detailed and comprehensive information.

Parents were described having a main role as information providers. While parent's presence and support are valuable their role as information providers has to be challenged (Smith & Callery, 2005). Since parental anxiety is strongly related to child preoperative anxiety they cannot be assumed to be able to respond to their needs (Kain et al., 1996; Kain, Mayes, Weisman, & Hofstadter, 2000). Interpreting children's messages in this study highlights the importance of supporting their need to process information to understand what is going to happen. Understanding relates to learning processes (Illeris, 2009; Kolb, 2014; Marton, 1997; Mezirow, 2009), and children have to construct their own understanding about anaesthesia and surgery to be prepared for the expected procedures.

The confident children expressed the importance of receiving information in good time which indicates the significance of giving children time to process the information provided. In a previous study (Lööf, Liljeberg, Eksborg, & Lönnqvist, 2017) children's and parent's perioperative level of knowledge was compared after receiving either interactive web‐based information or traditional brochure material. Interactive web‐based information was associated with a better level of knowledge in both children and parents. The actual interactive website was to a high extent used by the confident children in this study. In another study, the interactive website was analysed based on a pedagogical framework (Lööf, Andersson‐Papadogiannakis, Karlgren, & Silén, 2018). The analysis showed that an interactive website offer possibilities for children to learn by building on their own curiosity and interest, identification with others experiences and by getting answers related to their concerns.

The results from this study and previous research reporting on children's preoperative anxiety call for extensive changes. Taking the children's perspectives into account, it is time to re‐evaluate existing perioperative structures, the content and configuration of the preparation provided as well as to scrutinize established communication strategies.

### 4.1 Strengths and limitations

One limitation in this study was that children were recruited from the same healthcare setting, were relatively healthy and undergoing routine surgeries. Another limitation was the imbalance of gender which is explained by the type of surgeries performed during the week for data collection. Although the number was small, the qualitative approach provides deep insights of children's perspective. The included children represented a wide variation of ages, developmental levels, previous experiences of hospitalization, anaesthesia and surgery, reflecting the reality and the diversity of children seen in a paediatric perioperative setting. The research process and context have been thoroughly described to enable transfer to other contexts.

## 5 CONCLUSIONS

Anaesthesia and surgery are landmark events in children's lives affecting them both in a short and long‐term perspective. Children perceive the perioperative process with all their senses and their perspectives and interpretations differ from healthcare providers. Children's perspectives provide valuable insights for the improvement of perioperative preparation programmes as well as for healthcare providers interaction with children during the perioperative process. Confident children experienced comprehension of the situation, were well prepared and described the interaction with the healthcare providers as satisfactory. The opposite was the case with children expressing fear for the situation. Receptivity and responsiveness, as well as recognition of children's need to process vital information to reach understanding, are significant factors to establish trust and confidence in the highly technological perioperative environment.

### 5.1 Relevance to clinical practice

The perioperative processes takes place in a fast pace in a highly technical environment characterized by strictly defined routines performed, with high demands on effectiveness and production. Children's possibilities for preparation and learning as well as their availabilities for interaction with healthcare providers are considerably decreasing in this context. Based on the results from this study, showing the risk for children's needs and perspectives to be neglected, we would argue it is time to consider new approaches when meeting children in the perioperative setting. The preoperative meeting with children and parents constitutes an unique opportunity for information and communication which we claim must be mandatory and classified equal to other preoperative preparations. To optimize the opportunities of this meeting, we further suggest it to be extended to more than a collection of medical information and include inquiring into children's individual level of knowledge, need for preparation and learning as well as a follow‐up of their understanding and trust. Participation of healthcare professionals with complementary paediatric expertise and experience is thus necessary. Extensive knowledge about children's cognitive development and reactions during stress as well as training of skills to interpret children's different expressions are compulsory prerequisites to succeed. The perioperative process is a period of continuing activity. Even though children are prepared for the expected procedures, they most likely will be exposed to new, unexpected experiences highlighting the importance of not restricting children's need of information and preparation to a single event. A continuous process signified by individualized adjustments and flexibility recognizing that children need to process the information received to fully understand and be prepared has to be provided (Lööf et al., 2018).

We argue that to improve paediatric perioperative care, it is necessary to respect, consider and include children's perspectives. To optimize children's preparation for anaesthesia and surgery and to avoid this process only becoming an additional source of information the development and conformation have to be conveyed with the consideration of children's learning processes.

## CONFLICT OF INTEREST

The authors have no conflicts of interest to declare.
