# Supplementary material for: Children's own perspectives demonstrate the need to improve paediatric perioperative care
Source: Nurs Open. 2019 Jul 18;6(4):1363–71. doi: 10.1002/nop2.332 (PMC6805266; doi:10.1002/nop2.332)
Supplement: Supplementary file 1 [file NOP2-6-1363-s001.docx]

## Supplementary Appendix 1

# Interview guide

The semi-structured interviews followed an interview guide based on areas about children’s experiences of hospitalization, perioperative procedures and need for preparation

(1-5).

## Areas for the interviews:

### Information and preparation

### Perioperative procedures

### Anesthesia and surgery

### Pain and pain management

### Timing of the different perioperative phases

### Hospital environment

### Family support

The interviews began with an open and general conversation about the child’s interests and daily activities which gave the interviewer an opportunity to distract the child and individualize the interview technique. The interviews proceeded with close-ended questions as *“Have you eaten anything today”?* to help the child to begin to engage in the interview process and assist in identifying openings for additional questions (6). To provide further access to children´s views, interpretations of events, understanding and experiences of processes the interviews continued with open-ended questions where children were encouraged to use their own words (6, 7). All questions were adapted to the individual child and to different cognitive developmental levels (6-8).

To explore children´s experiences of the perioperative procedures, they were asked:

- What do you think are children´s thoughts in association with hospitalization, anesthesia and surgery?

And depending on age asked:

- If you were to tell a friend about hospitalization, anesthesia and surgery, how would you describe it? (3-6 years).
- If one of your friends was about to be operated on, how would you explain about anesthesia and surgery? (7-11 years old).
- What was your experience of hospitalization, anesthesia and surgery (12 years and older) (9).

The interviewer was actively engaged in the conversation and asked follow-up questions as; *“what do you mean?”, “can you explain?”, “can you tell me a little bit more about that?”, “where have you learned that?”* (9).

# References

1. Fortier MA, Chorney JM, Rony RY, Perret-Karimi D, Rinehart JB, Camilon FS, et al. Children's desire for perioperative information. Anesth Analg. 2009;109(4):1085-90.

2. Jaaniste T, Hayes B, von Baeyer CL. Providing children with information about forthcoming medical procedures: A review and synthesis. Clinical Psychology: Science and Practice. 2007;14(2):124-43.

3. Coyne I. Children's experiences of hospitalisation. Journal of Child Health Care. 2006;10(4):326-36.

4. Pelander T, Leino-Kilpi H. Children's best and worst experiences during hospitalisation. Scand J Caring Sci. 2010;24(4):726-33.

5. Smith L, Callery P. Children's accounts of their preoperative information needs. J Clin Nurs. 2005;14(2):230-8.

6. Irwin LG, Johnson J. Interviewing young children: explicating our practices and dilemmas. Qual Health Res. 2005;15(6):821-31.

7. Kirk S. Methodological and ethical issues in conducting qualitative research with children and young people: a literature review. Int J Nurs Stud. 2007;44(7):1250-60.

8. Kortesluoma RL, Hentinen M, Nikkonen M. Conducting a qualitative child interview: methodological considerations. J Adv Nurs. 2003;42(5):434-41.

9. Nilsson S, Björkman B, Almqvist A-L, Almqvist L, Björk-Willén P, Donohue D, et al. Children’s voices–Differentiating a child perspective from a child’s perspective. Dev Neurorehabil. 2015;18(3):162-8.
